# MAYGEN: an open-source chemical structure generator for constitutional isomers based on the orderly generation principle

**DOI:** 10.1186/s13321-021-00529-9

**Published:** 2021-07-03

**Authors:** Mehmet Aziz Yirik, Maria Sorokina, Christoph Steinbeck

**Affiliations:** Institute for Inorganic and Analytical Chemistry, Friedrich-Schiller University, Lessing Strasse 8, 07743 Jena, Germany

**Keywords:** Constitutional isomer generation, Algorithmic group theory, Algorithmic graph theory, Chemical graph generation, Open-source software, CDK

## Abstract

The generation of constitutional isomer chemical spaces has been a subject of cheminformatics since the early 1960s, with applications in structure elucidation and elsewhere. In order to perform such a generation efficiently, exhaustively and isomorphism-free, the structure generator needs to ensure the building of canonical graphs already during the generation step and not by subsequent filtering. Here we present MAYGEN, an open-source, pure-Java development of a constitutional isomer molecular generator. The principles of MAYGEN’s architecture and algorithm are outlined and the software is benchmarked in single-threaded mode against the state-of-the-art, but closed-source solution MOLGEN, as well as against the best open-source solution PMG. Based on the benchmarking, MAYGEN is on average 47 times faster than PMG and on average three times slower than MOLGEN in performance.

## Introduction

Unconstrained isomer generation has received attention over the past decades as a means to assess the theoretically existing chemical space and as a hypothesis generator. Recently, the works of Jean-Louis Reymond and coworkers for the creation of the GDB-11 [1], GDB-13 [2] and GDB-17 [3] databases, enumerating all possible molecules with 11, 13, and 17 non-hydrogen atoms, respectively, in the molecular formula, have laid out the motivations for unconstrained isomer generation and the exploitation of its results in sufficient detail. Such molecular generation methods can be used as hypothesis generators in areas such as computer-assisted structure elucidation, but also to answer broader questions such as the exact size of a chemical space. Structure generators that produce constitutional isomers take a molecular formula as input, e.g., $\text {C}_{10}\text {H}_{16}\text {O}$, and enumerate or output all possible chemical structures that can be built with the given set of atoms in the molecular formula. The history of chemical graph generators reaches back to the 1960s and started with the DENDRAL project [4]. Their structure generator, CONGEN [5], was based on the substructures building blocks and dealt well with the overlapping substructures. Another structure generator substructure building blocks based was Assemble [6]. Chemical graph generators are based on mathematical theorems, especially the application of algorithmic group theory [7] and combinatorial algorithms [8]. MASS was a tool for the mathematical analysis of molecular structures and constructes molecules by generating their adjacency matrices [9] and SMOG [10] was the successor of MASS. Adjacency matrices include the edge multiplicity information for each atom pair in molecules.

Despite the long history of research on the theoretical and practical generation of chemical graphs, the number of publicly available algorithms and software for this purpose is still limited. The available generators [11] are ASSEMBLE [6], COCON [12], DENDRAL [4], LSD [13], MOLGEN [14], OMG [15], PMG [16], SENECA [17] and SMOG [10]. These generators and more details are described in [11]. For several decades, the closed-source, commercial structure generator MOLGEN, developed in C at the University of Bayreuth, marked the state of the art in terms of speed and completeness. Recognising the need for an open-source structure generator, Peironcely and colleagues [15] developed the Open Molecule Generator (OMG). OMG, however, is orders of magnitude slower than MOLGEN. Following OMG, a parallelized structure generator, PMG, was developed based on the OMG algorithm. The 452,458 isomers of $\text {C}_{10}\text {H}_{16}\text {O}$, for instance, are generated in only 3 s by MOLGEN 5.0, whereas MAYGEN 1.4 and PMG take 10 and 45 s, respectively. For more benchmarks, see “Results” section of the present manuscript.

In this work, we present the development of an open-source structure generator MAYGEN, a pure-Java constitutional isomer generator based on the principle of orderly generation described by Grund et al. [18]. We benchmark our method against the fastest available open-source solution PMG as well as against the closed-source, de facto gold standard MOLGEN. On average, MAYGEN is 47 times faster than PMG and three times slower than MOLGEN. In an old Arabic saying, “may” refers to a drop of water, and we hope that MAYGEN will be a good contribution to the field and trigger a surge in the development of improved and faster versions eventually rivalling the best closed-source solutions and thereby serving the scientific community. The complete MAYGEN code, as well as precompiled binaries, are available on GitHub.

## Methods

MAYGEN 1.4 generates constitutional isomers of a given molecular formula with an orderly graph generation algorithm from the field of algorithmic group theory. The principles are described in detail in [18]. We summarize them as following. A graph with *p* nodes, ${1,2,3, \ldots , p}$ has its symmetry group $S_{p}$. This symmetry group includes all the permutations of these nodes. However, for the case of coloured graphs, the nodes need to be partitioned (Eq. 1), in other words, nodes are grouped based on their colours, degrees and edges.

1 $$\begin{aligned} \lambda := (\lambda _{1}, \lambda _{2}, \ldots ) \; with \; \sum _{i}\lambda _{i} = n_{i} \end{aligned}$$

**Fig. 1:**
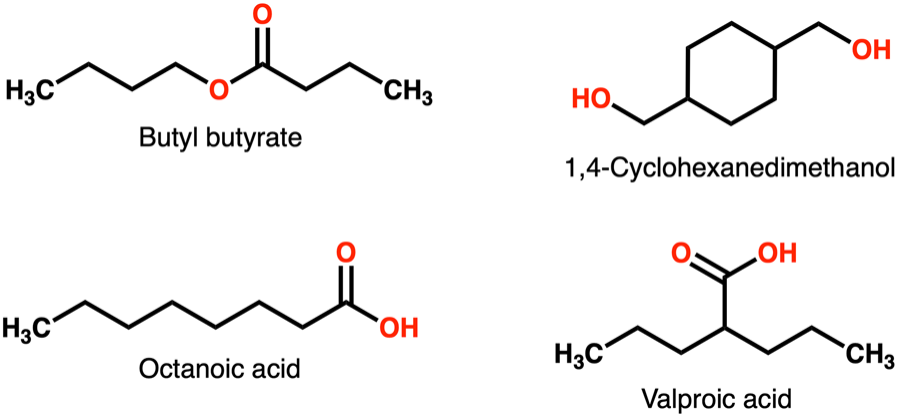
Four isomers of $\text {C}_{8}\text {O}_{2}\text {H}_{16}$. Atoms are coloured by their type

A molecule can be represented as a coloured graph. For 4 isomers of $\text {C}_{8}\text {O}_{2}\text {H}_{16}$ (Fig. 1), all atoms are coloured by their element types.

The atoms of $\text {C}_{8}\text {O}_{2}\text {H}_{16}$ can be partitioned in three groups as following: $\lambda ={2, 8, 16}$. For the case of this node partition, the symmetry group of 26 nodes, $S_{26}$, cannot be used since the nodes are coloured. In this case, a special type of symmetry group is applied, consisting of Young subgroups, that are the symmetry groups built based on the initial node partition (Eqs. 2 and 3).

2 $$\begin{aligned} n= & {} \bigcup \limits _{i}n_{i}^{\lambda } \, where \, n_{i}^{\lambda } = \left\{ \sum _{j=1}^{i-1} \lambda _{j}+1, \ldots , \sum _{j=1}^{i}\lambda _{j} \right\} \end{aligned}$$

3 $$\begin{aligned} S_{\lambda }:= & {} \left\{ \pi \in S_{n} | \forall i : \pi (n_{i}^{\lambda }) = n_{i}^{\lambda } \right\} \subseteq S_{n} \end{aligned}$$

In Eq. (2), these two summations give the minimum and maximum entries of the integer range. For the partition $\lambda ={2, 8, 16}$, its integer sets are:

$$\{1, 2\} \cup \{3, 4, 5, 6, 7, 8, 9, 10\} \cup \{11, 12, 13, 14, 15, 16, 17, 18, 19, 20, 21, 22, 23, 24, 25, 26\}$$

This symmetry group $S_{\lambda }$ is the direct product of Young subgroups permuting each atom type within its partition. In the case of $\text {C}_{8}\text {O}_{2}\text {H}_{16}$, the symmetry group of $S_{\lambda }$ is $S_{\{1, 2\}}* S_{\{3, 4, 5, 6, 7, 8, 9, 10\}}*S_{\{11, 12, 13, 14, 15, 16, 17, 18, 19, 20, 21, 22, 23, 24, 25, 26\}}$. The permutations of these symmetry groups only permute each element type within their groups, such as oxygens, carbons and hydrogens. The Young subgroups are then used for the construction of molecules’ automorphism groups (Eq. 4). These atom partitions and symmetry groups are the core part of the MAYGEN canonical test.

4 $$\begin{aligned} Aut(A) := \left\{ \pi \in S_{n} | A\pi = A \right\} \subseteq S_{p} \end{aligned}$$

MAYGEN’s construction of candidate structures consists of three distinct recursive tasks. First, the hydrogens are distributed to the heavy (i.e. non-hydrogen) atoms of the molecular formula. Then, the structures are generated in a block-wise manner, and finally, the canonical test avoids the generation of duplicate structures in an efficient and dynamic manner.

### Molecular formula check and hydrogen distribution

#### Graph existence check

Before calling the generator functions, there is a preliminary test for input molecular formulae. From graph theory, a degree list d can represent a graph with p nodes if the sum of all node degrees is equal or greater than $2*(p-1)$ and if the sum is an even number (Eq. 5) [18].

5 $$\begin{aligned} d = (d_{1},d_{2}, \ldots ,d{p}) \, \sum _{i=1}^{p} d_{i} \, is \, even \, and \, \sum _{i=1}^{p} d_{i} \ge 2*(p-1) \end{aligned}$$

A graph with *p* nodes should consist of at least $(p-1)$ edges. Since an edge is connected with two nodes in a graph, the sum of its node degrees should be equal to or greater than $2*(p-1)$.

#### Hydrogen distribution

For a given molecular formula, MAYGEN processes the hydrogens first and distributes them to all the other atoms in all possible ways since a hydrogen atom has a valence of 1 and can always have only one neighbour. The hydrogen distribution function takes two inputs, the atom partition and the number of hydrogens. The hydrogens are distributed in ascending order within each partition in order to avoid duplicates.

**Fig. 2:**
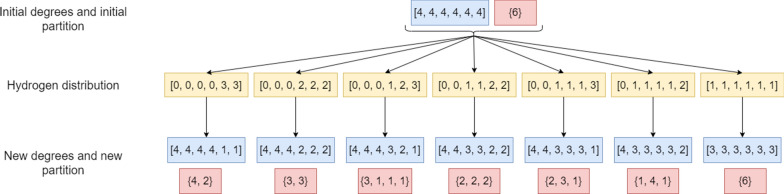
Illustration of the hydrogen distribution of C6H6 (in yellow) and its effect on the assigned atom valency (in blue) and on the atom partition (in red)

After the hydrogen distribution, the initial degrees and the initial partition are updated for each hydrogen distribution. For example, the non-hydrogen atoms from the molecular formula $\text {C}_{6}\text {H}_{6}$ have the initial respective degrees as [4, 4, 4, 4, 4, 4] and the initial partition {6}. There are 7 possible hydrogen distributions (Fig. 2) to these carbon atoms. After the hydrogen distribution step, the new lists of node degrees and partitions are used for the structure generation process. With the pre-hydrogen distribution, MAYGEN deals with a $6\times 6$ matrix instead of a $12\times 12$ matrix. The matrix size also has an impact on the canonical test since this test depends directly on the rows’ permutations. The hydrogen distribution code is available in the *hydrogenDistributor* Java class.

### Construction of candidate structures

**Fig. 3:**
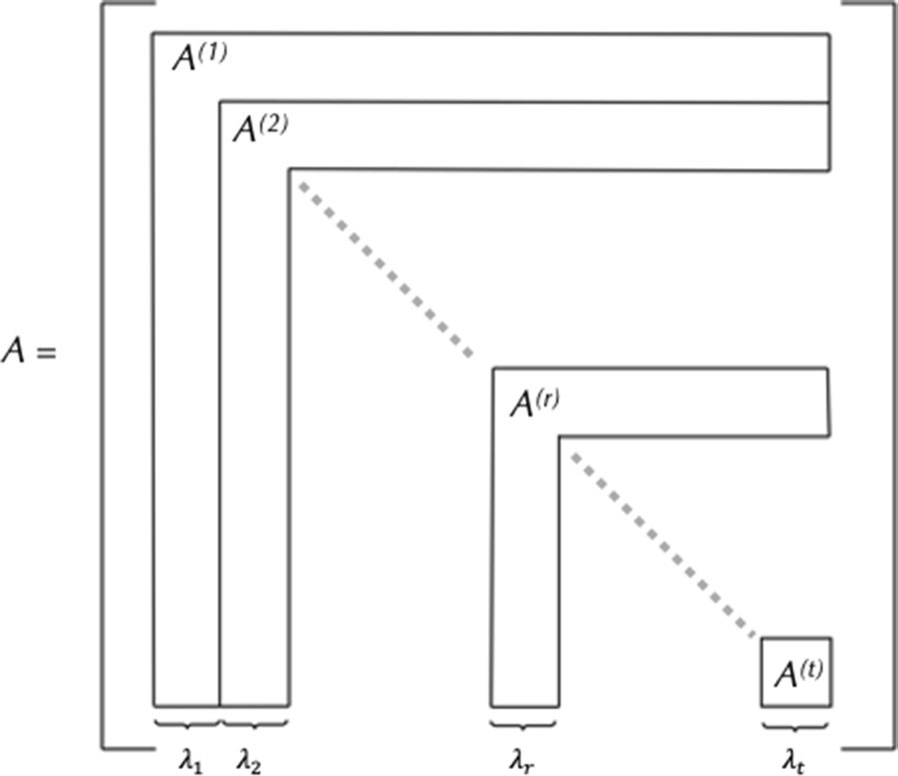
Block-wise representation of a matrix. Here, the matrix is split into parts based on the initial node partition with p entries. Image adapted from Grund et al. [18]

Once the molecular formula satisfies the graph existence criteria, the hydrogen distribution is performed to build a list of degrees. MAYGEN then starts the actual construction of candidate structures for each degree. The structures are represented by adjacency matrices in which each entry represents the edge multiplicity between the atom pairs. These matrices are built in a block-wise manner. The algorithm is based on the node degrees that correspond to the atom valences. The initial partition of the atoms, based on their element symbols, defines the blocks of the matrix (Fig. 3).

**Fig. 4:**
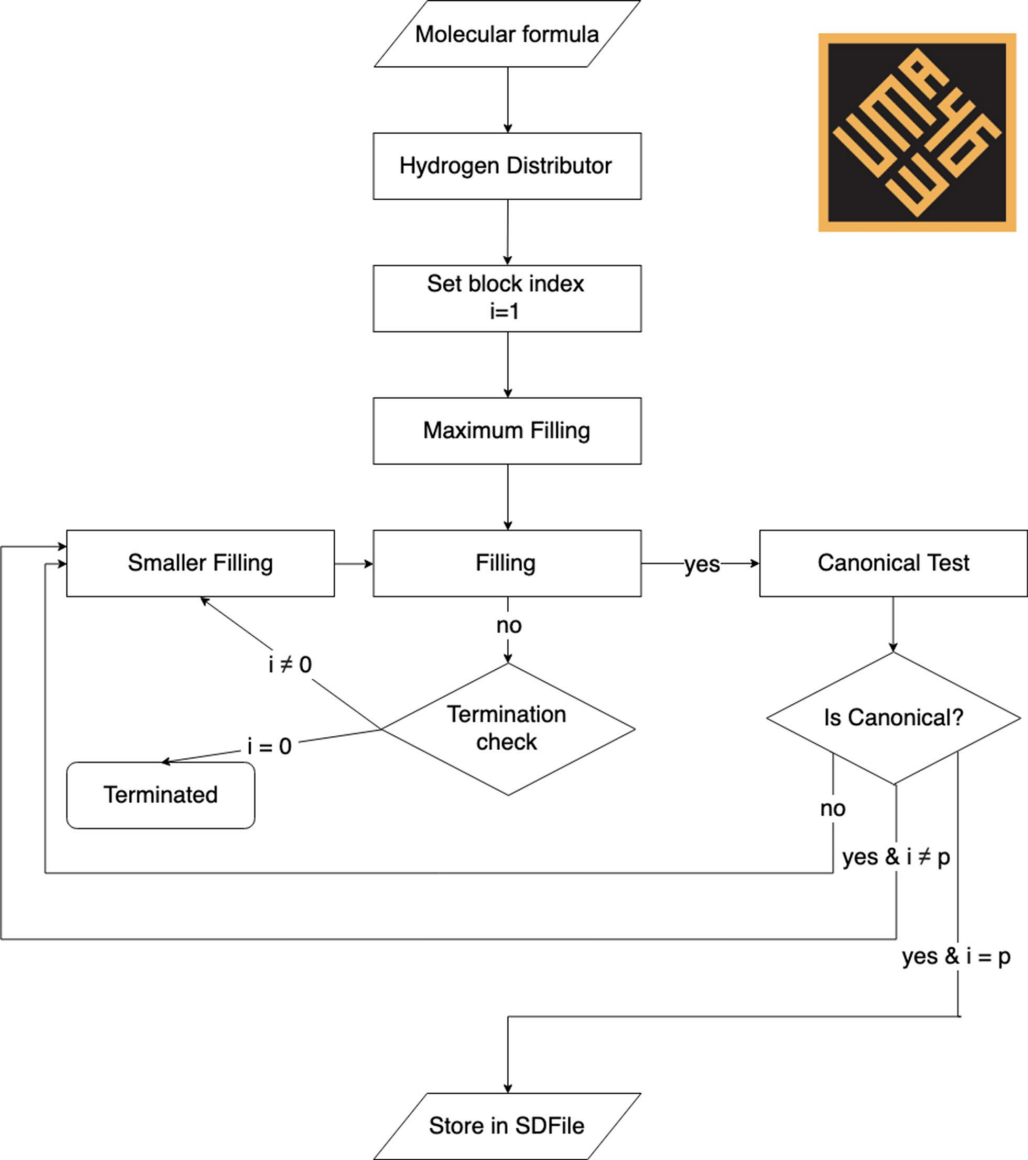
MAYGEN flowchart. The input formula includes p non-hydrogen atoms

With *p* being the number of atoms in the molecular formula without the hydrogens, an empty $p\times p$ matrix A is built. This matrix is filled in descending order starting with the maximal capacities and this is performed for each atom. The maximal capacity of an atom is calculated by decrementing its valence. For example, the valence of carbon is 4 and its maximal capacity is 3. Due to the diagonal symmetry of such matrices, only the upper triangular part needs to be filled. A canonical test, as described below, is performed once a block is filled. In a matrix, a block is defined as a number of rows and their transposes (i.e. columns). For example, a block between two indices 1 and 4 means the first 4 rows and the first 4 columns of the matrix. It needs to be noted that the canonical tests are performed without waiting for the whole matrix to be filled, which increases MAYGEN’s efficiency. This is the early boundary condition of the block-wise generation and avoids the construction of duplicate molecular structures. When the whole matrix is filled, it is written into the output SDF file, if such an option is selected at the beginning of the process. The algorithm then modifies the same input matrix A until there are no more possible changes. This is called the “build-and-forget method” [18]. The overall algorithm structure is explained in Algorithm 1 [18] and illustrated in Fig. 4.

Keeping the example of $\text {C}_{6}\text {O}_{2}\text {H}_{6}$, the initial valence vector is $v = [4,4,4,4,4,4,2,2,1,1,1,1,1,1]$, where the valences of each carbon atom are listed first, then the valences of each oxygen atom, and lastly the valences of all 6 hydrogen atoms. To optimize the process, the hydrogens are avoided in the further construction of the matrices by the hydrogen distribution step. Thus, the initial partition is $\lambda =\{6,2\}$ and the corresponding matrix is a $8\times 8$ matrix (built on 6 carbons and 2 oxygens).

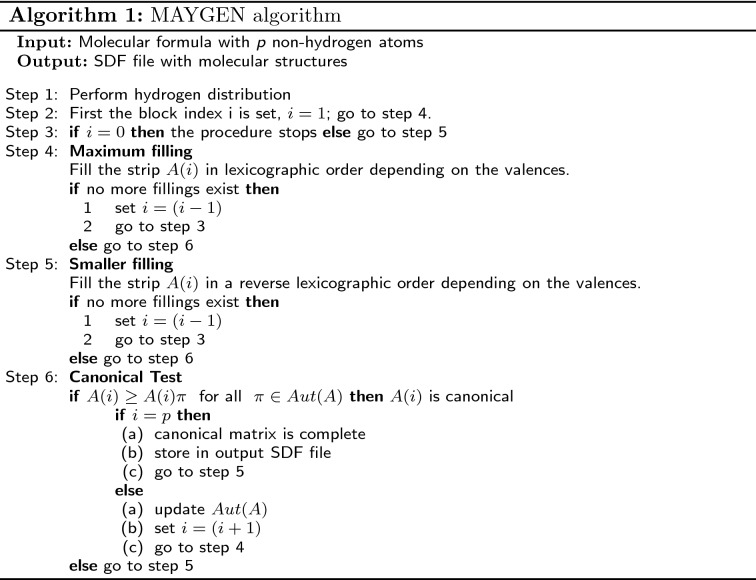



### Canonical test

The canonical test is the crucial part of the MAYGEN algorithm. In block-wise orderly structure generation, the early canonical testing avoids the construction of many duplicates. Overall, the purpose of the canonical test is the detection of the maximal matrix with respect to the given initial node partition.

6 $$\begin{aligned} A \ge A\pi \quad \forall \pi \in S_{\lambda } \end{aligned}$$

In the naive version of the canonical test, the matrix *A* is permuted for all the permutations of $S_{\pi }$ and its maximality is checked (Eq. 6). In the permuted matrices, $A_{\pi }$, their rows and entries are permuted. The original matrix *A* is compared with all the permuted matrices. Two matrices are compared row by row in a lexicographical order (Eq. 7).

7 $$\begin{aligned} \begin{aligned} A> A' : \Longleftrightarrow (a_{1,1},\ldots , a_{1,p},a_{2,1},\ldots , a_{2,p},a_{p,1},\ldots , a_{p,p} )\\ > (a'_{1,1},\ldots , a'_{1,p},a'_{2,1},\ldots , a'_{2,p},a'_{p,1},\ldots , a'_{p,p}) \end{aligned} \end{aligned}$$

In the block-wise orderly generation, only the rows within the blocks are compared.

#### Cycle transpositions

In the canonical test, the size of the symmetry group affects the run time of the algorithm. The initial partition is updated for each row during the test. Starting with the initial partition, with each row, the partitions are refined. The refinement process (Eq. 8) is explained below:

8 $$\begin{aligned} \lambda ^{(i)}= {\left\{ \begin{array}{ll} \underbrace{(1, ..., 1,}_\text {i-1}1,\lambda _{i}^{(i-1)} -1, \lambda _{i+1}^{(i-1)} ,...) &{} \text {if} \quad \lambda _i^{i-1} > 1\\ \underbrace{(1,...,1,}_\text {i-1}1,\lambda _{i+1}^{(i-1)} &{} \text {if} \quad \lambda _i^{(i-1)} = 1 \end{array}\right. } \end{aligned}$$

For $\text {C}_{3}\text {O}_{2}\text {H}_{4}$, the initial partition without hydrogens is {3,2}. Thus the partition list for all the rows are:

$$\begin{aligned} \begin{aligned} \lambda ^0&= \{3,2\}\\ \lambda ^1&= \{1,2,2\}\\ \lambda ^2&= \{1,1,1,2\}\\ \lambda ^3&= \{1,1,1,2\}\\ \lambda ^4&= \{1,1,1,1\} \end{aligned} \end{aligned}$$

These partition lists are used for the construction of the symmetry groups. By comparing the indices of two consecutive partitions, the cycle transpositions of symmetry groups are calculated. For partitions $\lambda ^{(i-1)}$ and $\lambda ^{(i)}$, the number of cycles is the $i$th entry in the former partition $\lambda _{i}^{(i-1)}$ (Eq. 9).

9 $$\begin{aligned} S_{\lambda ^{i-1}} = \cup _{j=i}^{\lambda _{i}^{i-1}}(i,j)S_{\lambda ^{i}},i=1,\ldots ,p-1 \end{aligned}$$

For example, the initial partition is {3, 2} and the refined partition for the first row is {1,2,2}. Here the number of cycle transpositions is 3 since the first entry of the former partition is 3. The cycle transpositions are (1,1), (1,2) and (1,3). These cycles are calculated row by row for all the partitions. The symmetry group of the molecule is calculated by the multiplication of all these cycles. The list of the partitions and their cycles are listed below:

$$\begin{aligned} \lambda ^0= & {} \{3,2\} \quad \lambda ^1 = \{1,2,2\} \quad Cycles: (1,1), (1,2), (1,3) \\ \lambda ^1= & {} \{1,2,2\} \quad \lambda ^2 = \{1,1,1,2\} \quad Cycles: (2,2), (2,3) \\ \lambda ^2= & {} \{1,1,1,2\} \quad \lambda ^3 = \{1,1,1,2\} \quad Cycles: (3,3) \\ \lambda ^3= & {} \{1,1,1,2\} \quad \lambda ^4 = \{1,1,1,1\} \quad Cycles: (4,4), (4,5) \end{aligned}$$

#### Calculation of automorphisms

In the canonical test, for a candidate matrix the corresponding automorphisms are calculated row by row. For the $i$th row of a matrix, the cycle transpositions $\varsigma _{i,j}$ are calculated based on the partitions $\lambda ^{(i-1)}$ and $\lambda ^{(i)}$. These cycle transpositions are used in the automorphisms search. All these cycles are multiplied in DFS manner with all the former automorphisms $\tau$ of the graph. This updated list of permutations are used in the canonical test of the matrix. For a graph with *p* nodes, its list of automorphisms until the $i$th row is:

10 $$\begin{aligned} F^{(i)} = \{\tau \in F^{(i-1)} | \tau * \varsigma _{(i,j)} \} \quad i<j<\lambda _{i}^{i-1} \end{aligned}$$

After the multiplication with all its cycles (Eq. 10), this updated list of automorphisms is used in the maximality check. If an automorphism is detected, that permutation is added to the automorphisms list, $F^{i}$. Thus, the automorphisms list is updated for each row until the row is in maximal form with respect to its partitions.

#### Maximality check

**Fig. 5:**
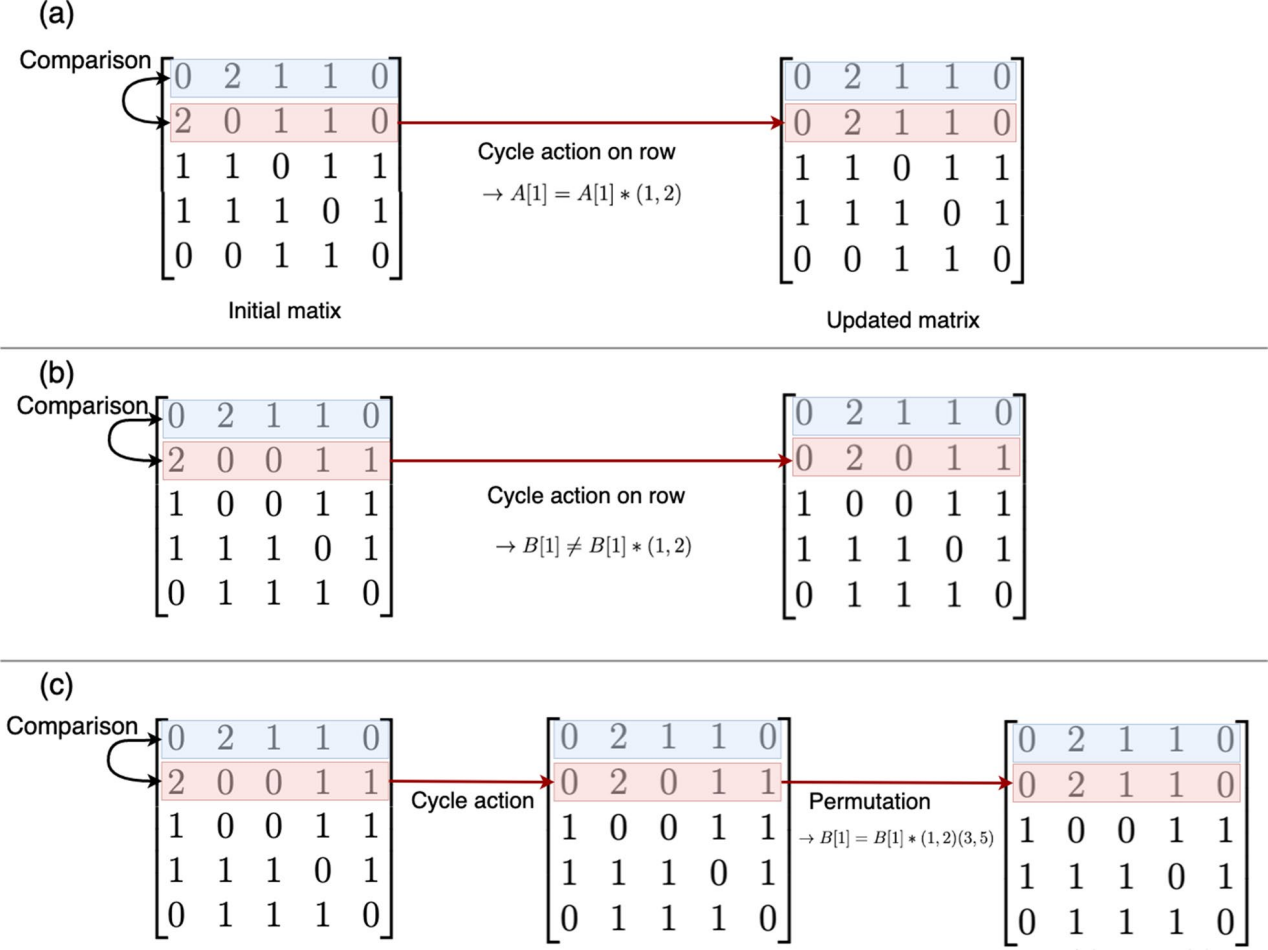
Maximality check. **a** A matrix A is permuted with a cycle transposition. The first and the second rows are identical after the permutation action. **b** A matrix B is permuted with a cycle transposition. The first and the second rows are not identical. **c** The canonical permutation of matrix B is given

For the maximality test of the $i$th row of a matrix, the row is compared with each permutation action in the automorphisms list. For each permutation, the original matrix *A* is permuted. Then, the $i$th rows of the original matrix and the permuted one are compared. These two rows are compared based on the $i$th atom partition. For an initial matrix *A*, as shown in Fig. 5a, with its partition $\lambda ^{(0)} = \{5\}$ and the refined partition $\lambda ^{(0)'} = \{1,4\}$, there are 5 cycle transpositions. One of these cycles is (1,2). To perform the maximality test, its first and second rows are compared (Fig. 5a).

In this example, the permutation (1,2) is an automorphism of the first row since it maps the row to itself in the adjacency matrix. Then this permutation is added to the list of automorphisms. However, in the case where a mapping with a cycle does not map the row to itself, a canonical permutation is needed. For an initial matrix *B* (Fig. 5b) with its initial partition $\lambda ^{(0)} = \{5\}$, its refined partition is $\lambda ^{(0)'} = \{1,4\}$, and there are 5 cycle transpositions for these partition. One of them is (1,2). To perform the maximality test, its first and second rows are compared (Fig. 5b).

Different from example A, in matrix *B* the first and second row are not identical after the cycle transpositions, and a canonical permutation is therefore needed. The canonical permutations are searched within the Young subgroups built with respect to the refined partition. In this example, the refined partition is $\lambda ^{(0)'} = \{1,4\}$. Thus, the symmetry group is $S_{\{1\}}*S_{\{2,3,4,5\}}$. For the canonical permutation search, only the permutations of the sets {1} and {2,3,4,5} are required. For the rows of matrix *B*, the canonical permutation is then (3,5), as depicted in Fig. 5c. Thus, (1,2)(3,5) is the automorphism of the first row and added to the automorphisms list for further testings.

In general, there are three criteria for updating the automorphisms list and for the maximality check:

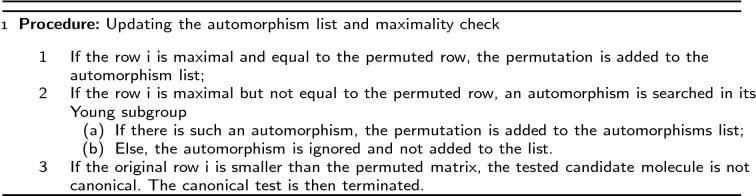



In the canonical test, if the row is canonical after testing all the permutations, the partition $\lambda ^{(i+1)}$ is built based on the $i$th row’s entries. After filling the entries of the $i$th row, i.e., adding bonds to the $i$th atom, the atom neighbourhoods are changed. Therefore the partition $\lambda ^{(i+1)}$ is defined based on the partition $\lambda ^{(i)}$ and the $i$th row entries. For matrix A and its refined partition $\lambda ^{(0)'}=\{1,4\}$, its partition first is updated with respect to the first row entries:

$$\begin{aligned}&\text {Refined partition }\lambda ^{(0)'}=\{1,4\} \rightarrow A[1]=[0 | {{2}} ,{{1, 1}}, {{0}}] \\&\quad \rightarrow \text {Updated partition }\lambda ^{(1)}=\{1,{{1}},{{2}},{{1}}\} \end{aligned}$$

The canonical test continues until the rows are in maximal form in lexicographic order. The automorphisms and partition lists are updated row by row.

#### Learning from canonical test

**Fig. 6:**
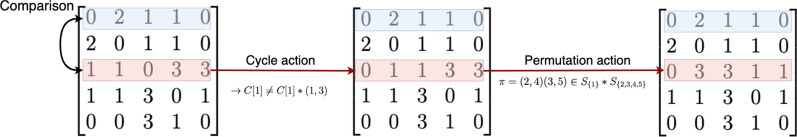
For a non-canonical matrix, detecting the entry indices makes it non-canonical

In case a molecule cannot pass the canonical test, there is still something to learn from the test. In the row by row comparison of the canonical test, when a row does not pass the test, the entry making it non-canonical is detected. As explained in Algorithm 1, if a block is not canonical, MAYGEN updates the matrix starting with its last entry in the block. However, with the help of the non-canonical matrix, the algorithm starts modifying the matrix from the entry making the matrix non-canonical. For a matrix *C* with its partition $\lambda ^{(0)}=\{5\}$ and the refined partition $\lambda ^{(0)'}=\{1,4\}$, there are 5 cycle transpositions. One of these cycles is (1,3). To perform the maximality test, its first and third rows are compared as shown in Fig. 6.

The permutation $\pi =(2,4)(3,5) \in S_{\{1\}}*S_{\{2,3,4,5\}}$ makes the third row bigger than the first row. Here the first entry making the row non-canonical is C[3, 4] in the matrix. Then the matrix construction continues with the indices [3, 4]. Using the learning from the canonical test, all the other non-canonical matrices are skipped.

### Connectivity test

**Fig. 7:**
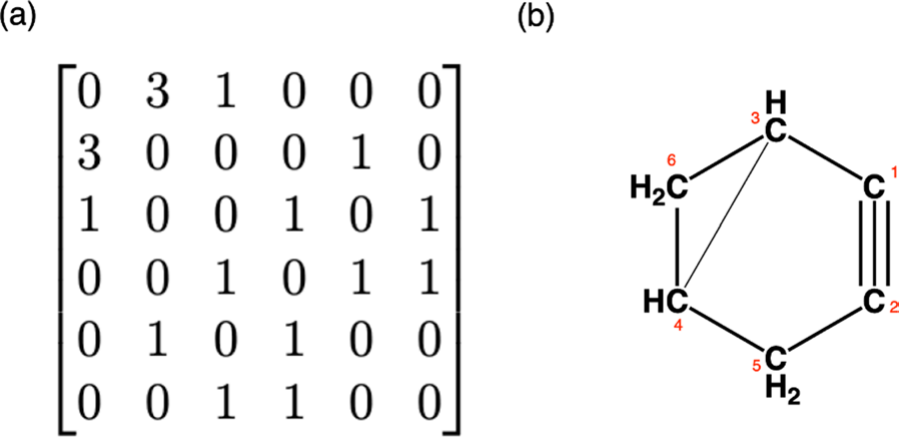
**a** The adjacency matrix of an isomer of $\text {C}_{6}\text {H}_{6}$. **b** An isomer of $\text {C}_{6}\text {H}_{6}$

The connectivity test of a graph is performed based on the neighbourhoods of all its nodes. The connectivity test starts with enumerating the nodes and setting this as the initial graph enumeration. The enumeration list is updated while checking the neighbour lists node by node. After detecting neighbours of a node, the labelling of the tested node and its neighbours from the graph enumeration list are stored. The minimum value of this set is given as the smallest index of the neighbourhood. This smallest index value is used for updating the list of graph enumeration. The test is terminated once all the nodes have the same label or all the nodes are re-labelled. For example, the connectivity test is performed for an isomer of $\text {C}_{6}\text {H}_{6}$ represented by the adjacency matrix A (Fig. 7a) with its initial node enumeration (labels) {1, 2, 3, 4, 5, 6} (Table 1).

**Table 1:** The connectivity test for an isomer of $\text {C}_{6}\text {H}_{6}$ represented by matrix A (Fig. 7a)

Node index	Neighbors	Former label	Minimum label	Enumeration
1	{1,2,3}	{1,2,3}	1	{1,1,1,4,5,6}
2	{2,5}	{1,5}	1	{1,1,1,4,1,6}
3	{3,4,6}	{1,4,6}	1	{1,1,1,1,1,1}

**Fig. 8:**
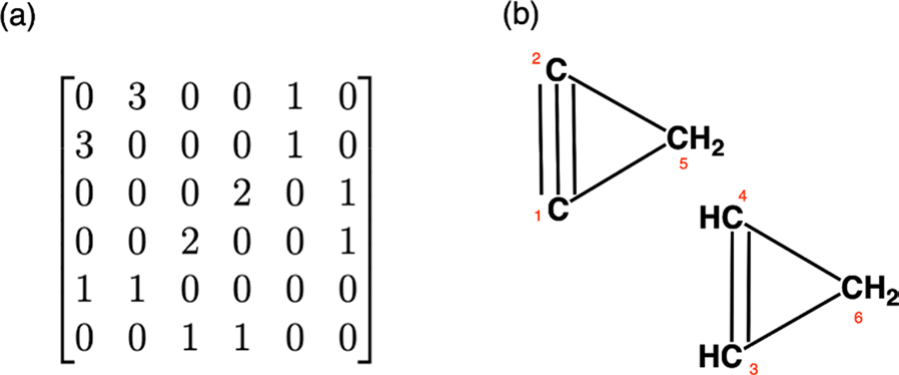
**a** The adjacency matrix of an isomer of $\text {C}_{6}\text {H}_{6}$. **b** A disconnected molecule with formula $\text {C}_{6}\text {H}_{6}$

The matrix *A* (Fig. 7a) is connected since the smallest node label for each tested node is 1 and its last node enumeration list includes only 1s. Thus there is only one component whose smallest index is 1 (Fig. 7b). For a disconnected chemical graph represented by the adjacency matrix *B* (Fig. 8a) with its initial node enumeration (labels) {1, 2, 3, 4, 5, 6}.

The matrix *B* represents a disconnected isomer of $\text {C}_{6}\text {H}_{6}$. This molecule has two components (Fig. 8b) with the indices $\varsigma _1= \{1,2,5\}$ and $\varsigma _2= \{3,4,6\}$. The first component $\varsigma _1$ is the first component with respect to its atom labelling. Here, components are compared with respect to their maximum index.

#### Learning from connectivity test

Similar to “Learning from canonical test”, there is still something to learn from the connectivity test if a molecule is not connected. In MAYGEN, the connectivity test is performed when a canonical matrix is complete. If a molecule is not connected, it is not stored in the output file and its first component needs to be detected. For example, the matrix *B* with Table 2, its first component is $\varsigma _1= \{1,2,5\}$. The maximum index of the first component identifies where the graph gets disconnected.

**Table 2:** The connectivity test for an isomer of $\text {C}_{6}\text {H}_{6}$ represented by matrix *B* (Fig. 8a)

Node index	Neighbors	Former label	Minimum label	Enumeration
1	{1,2,5}	{1,2,5}	1	{1,1,3,4,1,6}
2	{2,5}	{1}	1	{1,1,3,4,1,6}
3	{3,4,6}	{3,4,6}	3	{1,1,3,4,1,6}
4	{4,6}	{3}	3	{1,1,3,3,1,3}
5	{5}	{1}	1	{1,1,3,3,1,3}
6	{6}	{3}	3	{1,1,3,3,1,3}

In Algorithm 1, when a matrix is complete and stored in the output file, the generation process continues with the backward function. Here, the last index of the matrix is used as the input. However, with the “learning from connectivity test”, the algorithm continues with the last entry of the first component. For example, in matrix *B*, the first component is $\varsigma _1= \{1,2,5\}$ and the maximum index is 5. Thus, the graph gets disconnected after the last entry of the fifth row, *B*[5, 6] entry of the matrix *B*. All the other modifications on the matrix between its last entry [6, 6] and [5, 6] build only disconnected graphs. That is why the matrix modification process continues with the last entry of the first component. Learning from the connectivity test reduces the construction of disconnected graphs.

## Results

MAYGEN is written purely in Java and hosted on GitHub (see section Availability). The full source code, as well as pre-compiled binaries, are available for download. The code can be executed as follows:

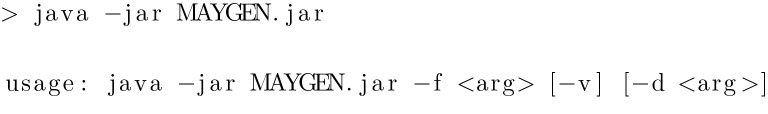



Generates molecular structures for a given molecular formula. The input is a molecular formula string, e.g. ‘C_2_OH_4_’. Besides this formula, the directory is needed to be specified for the output file.

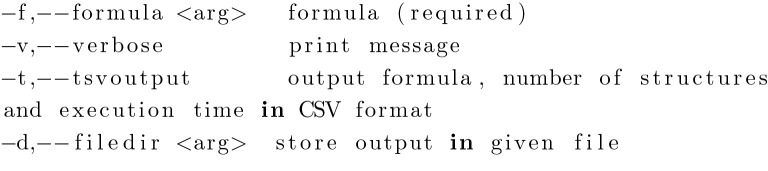



In order to generate constitutional isomers, the user needs to pass a molecular formula with the -f option:

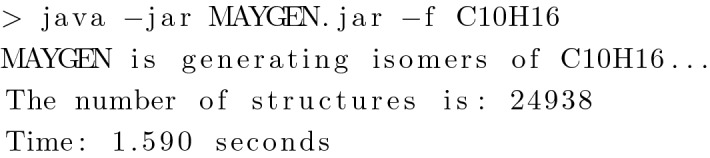



Alternatively, users who either want to contribute to the development or use the latest source code can clone the GitHub repository and build the MAYGEN binary using the Maven build environment.

For the purpose of this publication, MAYGEN was tested with randomly selected molecular formulae. The run times of MAYGEN, MOLGEN and PMG are compared in Table 4. The computational experiments were performed in single-threaded mode and without storing structures in an output file. PMG was tested against OMG and confirmed that even in single-threaded mode, PMG is faster. We used the latest version of Molgen, V 5, to be able to benchmark against larger numbers of molecular formulae. Molgen 3.5, which is faster than Molgen 5, is only available as a Windows GUI application and, to the best of our knowledge, cannot be run in batch mode. Furthermore, we do not own Windows license of Molgen 3.5. We did, however, manually run 10 formulae of the test version of Molgen 3.5 against the test version of Molgen 5 on the same Windows machine (Table 3).

**Table 3:** The number of structures and the run times are listed for MOLGEN 3.5 and MOLGEN 5.0 with a randomly selected 10 molecular formulae

Formula	# Structures	MOLGEN 3.5 runtime (s)	MOLGEN 5.0 runtime (s)	Ratio of runtimes
$\text {C}_{10}\text {H}_{15}\text {N}$	2,569,697	9	32	3.556
$\text {C}_{5}\text {HFIN}_{3}\text {O}$	2,737,786	7	38	5.429
$\text {C}_{7}\text {H}_{9}\text {NO}_{2}$	3,237,132	11	29	2.637
$\text {C}_{9}\text {H}_{12}\text {O}_{2}$	3,276,662	11	42	3.819
$\text {C}_{5}\text {H}_{6}\text {N}_{2}\text {O}_{3}$	4,513,867	11	40	3.637
$\text {C}_{9}\text {H}_{7}\text {N}$	2,521,767	6	42	7
$\text {C}_{5}\text {H}_{2}\text {BrClN}_{2}\text {O}_{2}$	5,211,489	9	50	5.556
$\text {C}_{8}\text {H}_{10}\text {O}_{3}$	3,869,189	13	44	3.385
$\text {C}_{7}\text {H}_{10}\text {O}_{4}$	1,428,242	5	16	3.2
$\text {C}_{7}\text {H}_{8}\text {O}_{4}$	2,709,647	9	31	3.445

The benchmark is performed without the aromaticity check

The comparison showed that Molgen 3.5 is about four times faster than Molgen 5 on average for these 10 tests. Different from MOLGEN 5.0 [14], PMG generates structures for additional valences of sulfur (S), phosphorus (P) and nitrogen (N) and therefore more molecules than MOLGEN or MAYGEN [15]. MOLGEN 5.0 uses the default lowest valences for N(3), S(2), and P(3), unless a user defines the higher valences. For all the results given in Table 4, MAYGEN generated the same number of structures as Molgen 5.0. Molgen has an aromaticity filter that filters out resonance structures of substituted aromatic molecules. This filter was deactivated with the **-noaromaticity** flag to achieve comparability. Since halogens are not defined in PMG, it does not generate structures with molecular formulae including Cl, F, Br, I.

**Table 4:** The number of structures and the run times are listed for MOLGEN 5.0, MAYGEN and PMG with a diverse set of molecular formulae

Formula	Structures (by MOLGEN and MAYGEN)	MOLGEN runtime (s)	Per structure (ms)	MAYGEN runtime (s)	Per structure (ms)	Structures (by PMG)	PMG runtime (s)	Per structure (ms)	Ratio MAYGEN/MOLGEN	Ratio PMG/MAYGEN
$\text {C}_{10}\text {H}_{15}\text {N}$	2,569,697	10	0.004	37	0.015	4,166,699	792	0.191	3.7	21.406
$\text {C}_{5}\text {HFIN}_{3}\text {O}$	2,737,786	10	0.004	21	0.008	N/A	N/A	N/A	2.1	0
$\text {C}_{7}\text {H}_{9}\text {NO}_{2}$	3,237,132	10	0.004	28	0.009	5,451,213	580	0.107	2.8	20.715
$\text {C}_{9}\text {H}_{12}\text {O}_{2}$	3,276,662	11	0.004	38	0.012	3,276,662	232	0.071	3.455	6.106
$\text {C}_{5}\text {H}_{6}\text {N}_{2}\text {O}{3}$	4,513,867	12	0.003	43	0.01	14,679,025	2643	0.181	3.584	61.466
$\text {C}_{9}\text {H}_{7}\text {N}$	2,521,767	13	0.006	22	0.009	5,076,949	484	0.096	1.693	22
$\text {C}_{5}\text {H}_{6}\text {P}_{2}\text {S}_{3}$	4,513,867	15	0.004	40	0.009	N/A	> 24 h	N/A	2.667	0
$\text {C}_{5}\text {H}_{2}\text {BrClN}_{2}\text {O}_{2}$	5,211,489	16	0.004	38	0.008	N/A	N/A	N/A	2.375	0
$\text {C}_{9}\text {H}_{7}\text {P}$	2,521,767	16	0.007	21	0.009	3,885,840	357	0.092	1.313	17
$\text {C}_{11}\text {H}_{10}$	3,614,427	20	0.006	39	0.011	3,614,427	204	0.057	1.95	5.231
$\text {C}_{8}\text {H}_{7}\text {NO}$	5,005,355	20	0.004	38	0.008	9,641,272	926	0.097	1.9	24.369
$\text {C}_{5}\text {H}_{2}\text {FIO}_{2}\text {P}_{2}$	5,211,489	20	0.004	37	0.008	N/A	N/A	N/A	1.85	0
$\text {C}_{8}\text {H}_{5}\text {NO}$	3,999,703	21	0.006	30	0.008	8,492,691	852	0.101	1.426	28.4
$\text {C}_{10}\text {H}_{16}\text {S}_{2}$	4,676,149	21	0.005	87	0.019	N/A	> 24 h	N/A	4.143	0
$\text {C}_{9}\text {H}_{10}\text {O}_{2}$	6,843,602	24	0.004	68	0.01	6,843,602	502	0.074	2.834	7.383
$\text {C}_{8}\text {H}_{7}\text {PS}$	5,005,355	24	0.005	38	0.008	51,262,825	7177	0.141	1.584	188.869
$\text {C}_{8}\text {H}_{5}\text {PS}$	3,999,703	25	0.007	31	0.008	44,966,952	6058	0.135	1.24	195.42
$\text {C}_{5}\text {H}_{5}\text {N}_{3}\text {O}_{2}$	9,390,618	26	0.003	71	0.008	70,007,293	15,845	0.227	2.731	223.17
$\text {C}_{10}\text {H}_{13}\text {N}$	7,122,614	27	0.004	78	0.011	12,328,415	1850	0.151	2.889	23.718
$\text {C}_{12}\text {H}_{20}\text {O}$	6,100,808	28	0.005	185	0.031	6,100,808	1160	0.191	6.608	6.271
$\text {C}_{9}\text {H}_{10}\text {S}_{2}$	6,843,602	29	0.005	68	0.01	347,718,450	79,415	0.229	2.345	1167.868
$\text {C}_{11}\text {H}_{8}$	4,442,438	30	0.007	46	0.011	4,442,438	296	0.067	1.534	6.435
$\text {C}_{10}\text {H}_{10}\text {O}$	7,288,733	30	0.005	71	0.01	7,288,733	502	0.069	2.367	7.071
$\text {C}_{5}\text {H}_{5}\text {P}_{3}\text {S}_{2}$	9,390,618	32	0.004	69	0.008	N/A	> 24 h	N/A	2.157	0
$\text {C}_{7}\text {H}_{6}\text {N}_{2}\text {O}$	10,504,307	37	0.004	77	0.008	41,261,882	5440	0.132	2.082	70.65
$\text {C}_{10}\text {H}_{8}\text {O}$	9,693,195	47	0.005	88	0.01	9,693,195	748	0.078	1.873	8.5
$\text {C}_{12}\text {H}_{14}$	11,451,841	52	0.005	158	0.014	11,451,841	864	0.076	3.039	5.469
$\text {C}_{10}\text {H}_{11}\text {N}$	14,778,466	57	0.004	140	0.01	27,530,678	3411	0.124	2.457	24.365
$\text {C}_{10}\text {H}_{14}\text {O}_{2}$	16,422,284	57	0.004	217	0.014	16,422,284	1645	0.101	3.808	7.581
$\text {C}_{5}\text {H}_{6}\text {BrClFIN}_{2}\text {O}$	23,955,660	57	0.003	248	0.011	N/A	N/A	N/A	4.351	0
$\text {C}_{11}\text {H}_{14}\text {O}$	20,354,040	76	0.004	250	0.013	20,354,040	1714	0.085	3.29	6.856
$\text {C}_{9}\text {H}_{11}\text {NO}$	25,895,621	86	0.004	239	0.01	46,139,031	6088	0.132	2.78	25.473
$\text {C}_{5}\text {HFIP}_{3}\text {S}_{2}$	22,825,473	97	0.005	178	0.008	N/A	N/A	N/A	1.836	0
$\text {C}_{7}\text {H}_{9}\text {Br}_{2}\text {Cl}_{2}\text {PS}$	26,610,607	109	0.005	452	0.017	N/A	N/A	N/A	4.147	0
$\text {C}_{12}\text {H}_{18}\text {O}$	28,140,012	114	0.005	551	0.02	28,140,012	3656	0.13	4.834	6.636
$\text {C}_{9}\text {H}_{12}\text {F}_{2}\text {I}_{2}\text {S}$	25,427,769	126	0.005	558	0.022	N/A	N/A	N/A	4.429	0
$\text {C}_{10}\text {H}_{12}\text {O}_{2}$	42,261,751	146	0.004	474	0.012	42,261,751	3692	0.088	3.247	7.79
$\text {C}_{9}\text {H}_{9}\text {NO}$	43,311,373	156	0.004	365	0.009	83,676,810	10,116	0.121	2.34	27.716
$\text {C}_{11}\text {H}_{12}\text {O}$	46,647,199	181	0.004	498	0.011	46,647,199	3818	0.082	2.752	7.667
$\text {C}_{12}\text {H}_{10}$	37,720,012	210	0.006	405	0.011	37,720,012	3107	0.083	1.929	7.672
$\text {C}_{9}\text {H}_{5}\text {NO}$	36,456,956	214	0.006	275	0.008	84,685,537	11,133	0.132	1.286	40.484
$\text {C}_{12}\text {H}_{4}$	16,079,924	215	0.014	366	0.023	16,079,924	3675	0.229	1.703	10.041
$\text {C}_{9}\text {H}_{7}\text {NO}$	49,865,161	218	0.005	407	0.009	105,236,547	11,983	0.114	1.867	29.443
$\text {C}_{12}\text {H}_{8}$	43,435,791	307	0.008	449	0.011	43,435,791	4147	0.096	1.463	9.237
$\text {C}_{12}\text {H}_{6}$	34,030,905	325	0.01	508	0.015	34,030,905	4169	0.123	1.564	8.207
$\text {C}_{11}\text {H}_{10}\text {O}$	79,818,477	348	0.005	765	0.01	79,818,477	6856	0.086	2.199	8.963
$\text {C}_{10}\text {H}_{4}\text {S}_{2}$	495,428,30	413	0.009	472	0.01	N/A	> 24 h	N/A	1.143	0
$\text {C}_{11}\text {H}_{10}\text {S}$	79,818,477	429	0.006	751	0.01	N/A	> 24 h	N/A	1.751	0
$\text {C}_{10}\text {H}_{8}\text {S}_{2}$	105,772,510	466	0.005	963	0.01	N/A	> 24 h	N/A	2.067	0
$\text {C}_{10}\text {H}_{6}\text {S}_{2}$	93,964,875	552	0.006	850	0.01	N/A	> 24 h	N/A	1.54	0

MAYGEN always generates the same number of structures as MOLGEN. Times for Molgen were determined with the **-noaromaticity** flag to achieve comparability. PMG generates more structures in some cases due to different valences of S, P and N, which is why the per molecule run time is also given in milliseconds (ms)

**Fig. 9:**
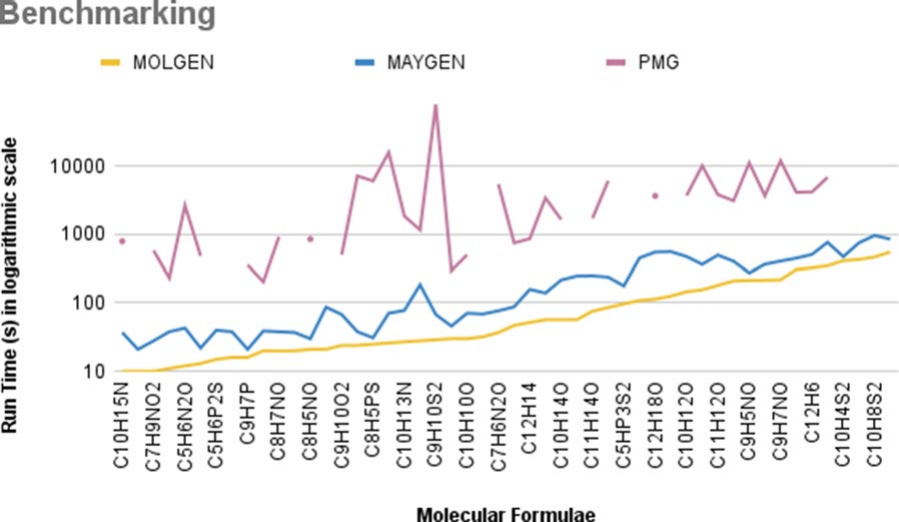
Times for structure generation runs with MOLGEN 5.0, MAYGEN and PMG for molecular formulae containing all allowed elements (carbon, hydrogen, oxygen, nitrogen, phosphorus, sulfur and halogens. The total run times (s) are plotted. For a fairer comparison, Fig. 10 shows the per-molecule run times

**Fig. 10:**
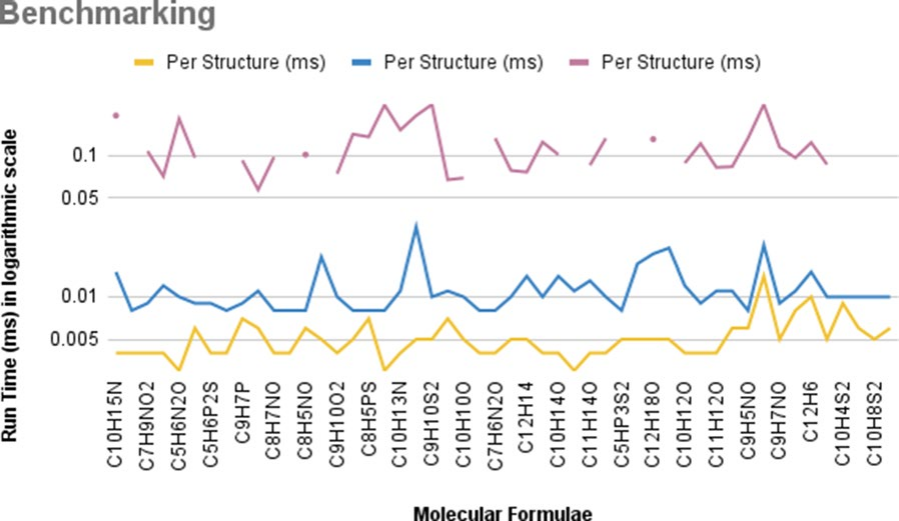
Times for structure generation runs with MOLGEN 5.0, MAYGEN and PMG for molecular formulae containing all allowed elements (carbon, hydrogen, oxygen, nitrogen, phosphorus, sulfur and halogens. Since PMG generates additional structures with higher oxidation states for N, S and P the run times (ms) for the construction of per molecule are plotted

For most structures containing all allowed elements, MOLGEN was slightly faster than MAYGEN and much faster than PMG (Figs. 9, 10); for carbohydrates and those containing additional oxygen, MAYGEN’s execution speed was comparable to that of MOLGEN. Since PMG does not generate structures for formulae with halogens, “N/A” is added to the result table. “> 24 h” is added to the result for the formulae for which PMG took longer than a day. These results are visualized with spaces in the plots (Figs. 9, 10).

### Limitations

MAYGEN is currently restricted to generate molecules with the lowest valence states of nitrogen, phosphorus and sulfur, and all testing and benchmarking was done under this boundary condition. This is no principle restriction—the algorithm will work with any given valence state—but the workflow logic of MAYGEN needs to be adapted to compute structures for higher valences of these elements.

## Future work

Being implemented in pure Java and with its code completely open, MAYGEN can be easily extended with additional functionalities and algorithmic improvements. The code availability through GitHub invites the scientific community to contribute to the further developments of MAYGEN. Obvious future work includes performance enhancements and the parallelization of the algorithm. Future implementations of MAYGEN will be parallelised. The lowest hanging fruit will be exploiting the built-in parallelism in the Java VM using multiple available cores. Here, trivial parallelism can be used by computing the isomers of different hydrogen distributions simultaneously. With 8 cores in the CPU of the senior author’s laptop and 18 cores in individual CPUs on our local compute cluster, significant speed gains can be achieved through this simple measure. The examples in our results Table 4 have between 2 and 74 hydrogen partitions, which yields plenty of space for further speed gains. Trivial parallelism can be pushed further by recent cloud orchestration schemes where containers can be seamlessly launched in large clouds, for example using the Google Container Engine. Here, the number of parallel computations X can be matched to fit the number of partitions precisely, leading to an approximate speed gain of X, ignoring the container provisioning and result collection. More elaborate non-trivial parallelisation schemes will be needed to push the boundary of computing with more heavy atoms in each molecular formula beyond the current 15–20 atom limit. The exponential explosion of the number of isomers in this region, will only allow for very moderate advances though. We also aim to integrate MAYGEN into the Chemistry Development Kit (CDK) [19] in the near future which will enable an easy integration of the molecular structure generator in other software programmatically. Furthermore, it is desirable that MAYGEN can use substructures in its input as building blocks, in order to include them as badlists or goodlists into the generation and therefore reduce the number of candidate structures to generate. This will enable its use in systems for computer-assisted structure elucidation (CASE) whose aim is to elucidate chemical structures from NMR and mass spectral data.

## Conclusion

In this manuscript we presented MAYGEN, an open-source constitutional isomer generator completely written in Java. MAYGEN generates constitutional isomer spaces exhaustively and avoids isomorphic structures during the generation using the principles of orderly canonical graph generation. We presented extensive testing of MAYGEN against two alternative solutions: MAYGEN outperforms the current best open source structure generator PMG by orders of magnitude, on average 47 times faster, and is only marginally slower, on average three times, than the fastest current state-of-the-art software MOLGEN. We expect MAYGEN to be a starting point for further developments in the area of chemical structure generation by the open source, open science community.

## Data Availability

Project name: MAYGEN, Project home page: https://github.com/MehmetAzizYirik/MAYGEN, Operating system(s): Platform independent, Programming language: Java, License: MIT.
